# Modeling the live-pig trade network in Georgia: Implications for disease prevention and control

**DOI:** 10.1371/journal.pone.0178904

**Published:** 2017-06-09

**Authors:** Esther Andrea Kukielka, Beatriz Martínez-López, Daniel Beltrán-Alcrudo

**Affiliations:** 1 Center for Animal Disease Modeling and Surveillance (CADMS), Department of Medicine & Epidemiology, School of Veterinary Medicine, University of California, Davis, California, United States of America; 2 Food and Agriculture Organization, FAO Budapest, Hungary; Missouri Botanical Garden, UNITED STATES

## Abstract

Live pig trade patterns, drivers and characteristics, particularly in backyard predominant systems, remain largely unexplored despite their important contribution to the spread of infectious diseases in the swine industry. A better understanding of the pig trade dynamics can inform the implementation of risk-based and more cost-effective prevention and control programs for swine diseases. In this study, a semi-structured questionnaire elaborated by FAO and implemented to 487 farmers was used to collect data regarding basic characteristics about pig demographics and live-pig trade among villages in the country of Georgia, where very scarce information is available. Social network analysis and exponential random graph models were used to better understand the structure, contact patterns and main drivers for pig trade in the country. Results indicate relatively infrequent (a total of 599 shipments in one year) and geographically localized (median Euclidean distance between shipments = 6.08 km; IQR = 0–13.88 km) pig movements in the studied regions. The main factors contributing to live-pig trade movements among villages were being from the same region (i.e., local trade), usage of a middleman or a live animal market to trade live pigs by at least one farmer in the village, and having a large number of pig farmers in the village. The identified villages’ characteristics and structural network properties could be used to inform the design of more cost-effective surveillance systems in a country which pig industry was recently devastated by African swine fever epidemics and where backyard production systems are predominant.

## Introduction

Movement of live animals plays an important role in the spread of infectious diseases [1]. For this reason, a better understanding of the live animal movement patterns and the ability to promptly trace them in emergency situations have been recognized as key to prevent, early detect, rapid control and even predict disease outbreaks [2–4].

However, scarce information is available about live animal movements in countries where backyard production is predominant [5]. With premises and animals often unregistered and no movement of animals recorded, data collection in these areas is generally limited and drivers for trade are mostly unknown and can suddenly change in response to market fluctuations or the appearance of infectious diseases. An example of such scenario is the pig industry in the country of Georgia, in the Caucasus region, where the majority of the swine production (over 90%) can be classified as backyard and where very scarce information is available on pig trade patterns. This lack of information in combination with other factors, such as the presence of wild boar populations and illegal trade of pigs and pig products contributed to the difficulties to control the African swine fever (ASF) epidemic in the country when it was introduced in 2007, facilitating its further spread to the Russian Federation and the rest of the Caucasus region [6, 7].

Backyard production systems, usually characterized by low technification and scarcity of biosecurity practices, can play an important role in disease transmission and maintenance, particularly in diseases such as ASF, where no vaccine is available [8, 9]; thus, a better knowledge of the pig trade patterns of this type of production will help to design more cost-effective disease prevention and control programs to make the swine industry more resilient [10].

In this study, we characterized the structure, contact patterns and main drivers of the backyard pig trade network in Georgia with the aim to inform and facilitate the design of risk-based and more cost-effective disease surveillance and control programs in the country. Moreover, methods could be easily implemented in other backyard predominant production systems in the region.

## Materials and methods

### Study region and sample selection

Georgia is located in the Caucasus region, bordering to the Russian Federation, Turkey, Armenia, Azerbaijan and the Black Sea. Currently, the country is divided in 9 regions, 2 autonomous republics and 76 municipalities. The total population in Georgia is 3.7 million people, from which approximately 1.6 million live in rural areas. Georgian values and farming culture have been strongly influenced by a communist background until the independence from the Soviet Union in 1991. Most farms in soviet Georgia were state-run, leaving a meager private infrastructure behind that lead to a loss of opportunity to develop private farming. The Georgian pig market, once proliferous (1,173 thousand heads in 1985), has been decreasing at an irregular rate for the last 30 years [11], accounting for two main inflexion points. The first steep decrease occurred on 1991, after the independence from the Soviet Union; the second decline occurred with the 2007 ASF epidemic into the country, which decimated the pig population to official numbers as low as 86.4 thousand heads in 2008 [11] and caused an increase of pork produce prices of 273%, from 2006 to 2012 [12]. Nowadays, farming cooperatives are slowly thriving and reshaping the Georgian farming system.

**Fig 1:**
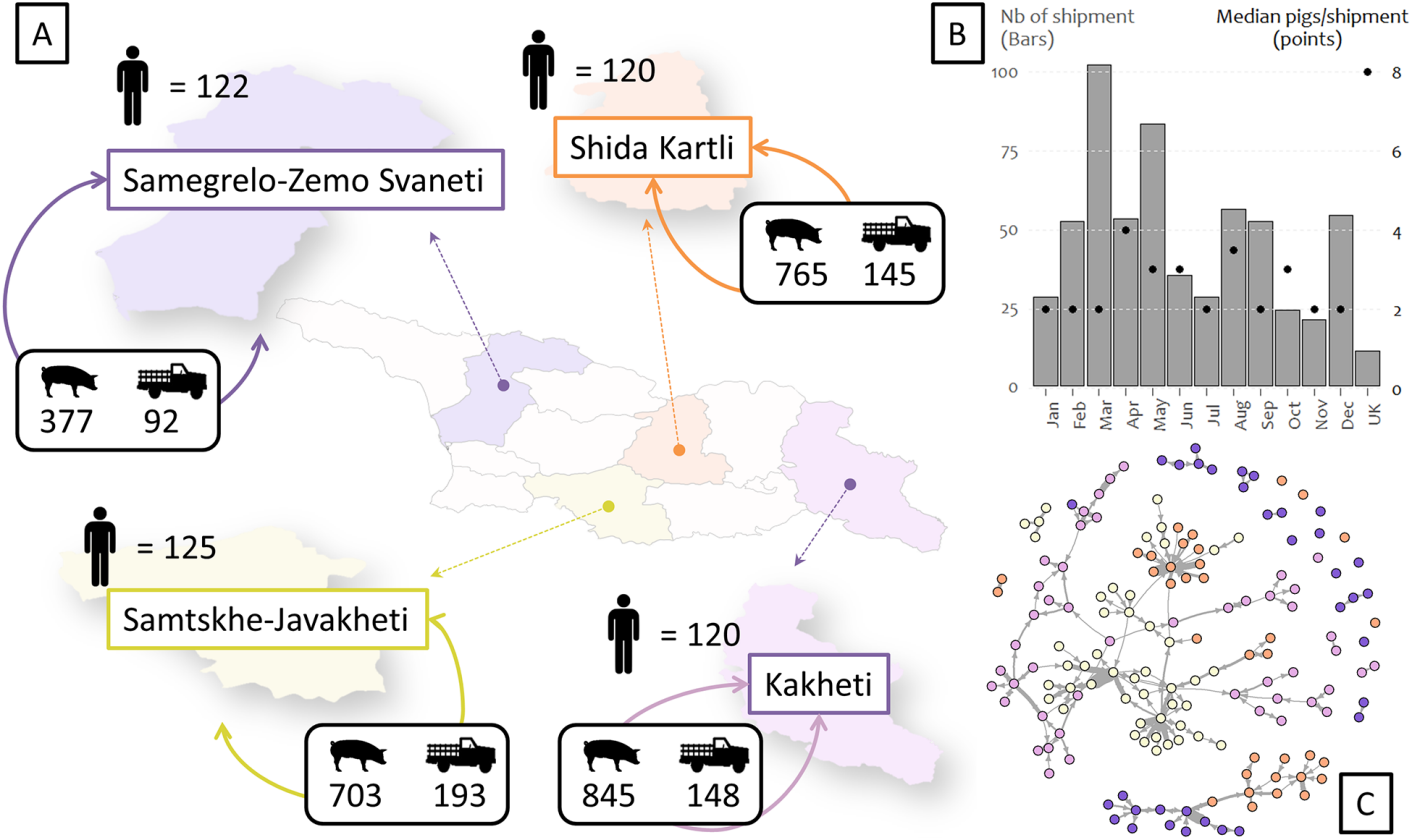
A: Number of farmers (n = 487) interviewed per region (n = 4), number of pigs shipped and number of shipments within region in Georgia, as reported by farmers during a 12 months’ scope questionnaire; B: Monthly frequency of shipments (bars) and median number of pigs transported per shipment (dots), C: Network visualization. Colors of the regions in the map correspond to colors of the network. *UK* = Unknown; Pig image = Total number of pigs shipped within region; Truck image = Total number of shipments within region.

This study was conducted in four pig rearing regions of Georgia (namely, Kakheti, Shida Kartli, Samtskhe-Javakheti, and Samegrelo Zemo-Svaneti). Region selection criterion was presence of a veterinary association. A total of 26 municipalities and 168 villages were conveniently sampled (Fig 1). The target population in the study was pig farmers located in diverse settings (i.e., big towns and small villages both in valley and mountainous areas).

### Questionnaire design

A semi-structured questionnaire was designed by FAO veterinarians, written in English and translated into Georgian and Russian (S1 Table). Prior to the administration of the questionnaire, veterinary services from the Georgian National Food Agency were informed about the project and oral permission from them was obtained. At the time of the design and implementation of the survey, FAO followed the principles of the declaration of Helsinki and the Belmont report [13]. The UC Davis Institutional Review Board (IRB) Administration determined that this project was exempt from the requirement for IRB review. The exemption criteria are found at “45 CFR 46.101(b)(2)–U.S. Code of Federal Regulation, Protection of human subjects”. All selected farmers were informed of the study purpose, the volunteer and anonymous nature of the participation in the interviews and the possibility of dropping from the study at any time. A pilot study (n = 30) was carried out by local veterinarians (not trained on questionnaire administration) across eight municipalities in Kakheti region in order to assess the performance of the questionnaire. The questionnaire was divided into six sections: farm demographics, farm health status, management and home slaughter practices, pig trade, biosecurity and ASF awareness. All questions referred to the 12 months prior to the implementation of the questionnaire. Personal interviews were held in Georgian or Russian between September and November 2012 and administered by trained private veterinarians belonging to regional veterinary associations. Estimated duration was 20–30 minutes. Data collection was done on paper and posterior data entry was carried out in Excel. Missing data and contradictory responses were checked via telephone calls with the farmers and corrected.

### Network construction and data analysis

A directed network was constructed from the data collected within the pig trade section of the questionnaire. Data regarding origin and destination of the shipments was collected at the village level; thus, we defined villages as nodes, and the shipment of at least one live pig from one village to another as edges or ties. Due to the village level structure of our network data, quantitative variables from the questionnaire (asked at the farmer’s level) were aggregated to village level according to their mean or median value; whereas binomial variables (also asked at the farmer’s level) were collapsed within village as per absence/presence (at least one farmer per village).

The network presented is incomplete, as only four out of nine regions of the country were included. Shipments involving regions excluded in our survey (n = 18 shipments) were omitted from the calculation of both network level statistics and centrality measures. Within the four study regions, 45 villages that were not covered in the survey had traded with villages that were covered in the survey, yielding missing data observations (i.e., no questionnaire was obtained from those 45 villages).

Descriptive statistics, basic network characteristics and measures of central tendency and dispersion were computed for collected variables. Shipment distance (Euclidean distance, in km), shipment size (i.e., number of pigs shipped), monthly shipment frequency and centrality measures (indegree, outdegree and betweenness) were computed.

An exponential random graph model (ERGM) was used to model the probability of pig trade between villages as a function of both village characteristics and network structure. Unlike traditional regression models, ERGM provides an ideal statistical framework for network analysis that allows to account for dependence in tie formation as trade between two villages usually is influenced by both, the characteristics of other villages and the structure of the network itself (i.e., probability of trade between villages A and B partially depends on different attributes from other villages different than A and B) [14].

In order to work with ERGM, a simplified weighted network (i.e., a network where multiple edges and loops are removed and the number of shipments is used as edge weight) was created from the original directed network and missing values of villages attributes were imputed by multivariate imputations by chained equations using the predictive mean matching method [15].

Model building was conducted following a manual, three step, forward elimination process, using AIC for model selection (lower AIC values were preferred) [16, 17]. First, a null model (m0, Erdos-Renyi model) with “edges” capturing the density of the network was run and used as baseline to compare further models. Then, edge and node level predictors (exogenous, dyad independent terms [14, 18]) were added one by one to the null model and evaluated individually (m1, univariate analysis) [19]. Edge and node level predictors tested in the model were: altitude, presence/absence of suspected ASF in the village, type of backyard production system (i.e., free range/enclosed), number of farmers per village, income (village median of the percentage of income derived from pig farming), usage of a live animal market to trade live pigs by at least one farmer in the village, usage of a middleman to trade live pigs by at least one farmer in the village, total number of pigs in the village, average farm size per village, median percentage of produced pork that goes under heat treatment per village, and region. Edge and node level predictors are treated by the model using a non-stochastic logistic regression (i.e., assumption of independent observations). Finally, structural attributes (endogenous, dyad dependent terms) such as mutuality, gw*-type terms (gwindegree, gwoutdegree, gwdsp, gwesp), isolates and cyclical ties [19] were included into the model (m2). A glossary of ERGM-related terms is presented in Table 1. We also tested the hypothesis of uniform homophily between regions, as local trade would be expected in the dominant backyard pig farming system present in Georgia. A positive and significant coefficient for this uniform homophily term would suggest a higher likelihood of trade within region than between different regions. A negative and significant value for this coefficient would suggest that it is more likely to trade between regions than within the same region. Other hypothesis tested was that having at least one farmer in the village trading thorough live animal markets or middlemen yield to a higher probability of trade in that village. In this case, a positive and significant coefficient for each of those two variables would suggest such a premise. The model with structural attributes is estimated using a stochastic process based on MCMC under the premise that the probability of trade between two villages does not depend only on villages’ characteristics, but also on the dynamics of the network structure as a whole [14, 20]. Therefore, the use of structural attributes aims to more realistically represent the actual trade network.

**Table 1:** Glossary table of ERGM related terms.

Term	Definition
Degeneracy	When the fitted model suggests unlikely probabilities such as zero (empty graph, where no ties occur) or one (complete graph, where all possible ties occur) to the estimates of the model. These suggested probabilities do most likely fail to correctly fit the observed model; thus, the maximum likelihood estimator algorithm does not converge or offers an erratic solution. [14, 21]
Dyad dependent terms (endogenous)—structural predictors	These terms imply that ties between villages depend on attributes of the network as a whole, instead of depending only on the individual attributes of the villages [14, 18]. Some examples are GWD, GWDSP, and GWESP (see below).
Dyad independent (exogenous) terms—edge and node level predictors	These terms imply that ties between villages depend only on the individual attributes (characteristics/qualities) of the villages themselves [14, 18].
Geometrically weighted degree (GWD)	Structural predictor that negatively weights high degree nodes, and positively weights low degree nodes [22].
Geometrically weighted dyad-wise shared partnership (GWDSP)	Structural predictor that measures how likely two villages (A and B) that have another village (C) in common are to have another village (D) in common, regardless of whether there is a tie that links A and B or not [23].
Geometrically weighted edgewise shared partnership (GWESP)	Structural predictor that measures how likely two villages (A and B) that have another village (C) in common are to have another village (D) in common, when there is a *tie that links* A to B [23].
Edge-wise shared partners statistic	A statistic that explains the tendency of villages that trade amongst themselves to also trade with multiple shared villages [20].
Minimum geodesic distance statistic	A statistic that represents the shortest number of shipments needed to connect two villages [24]

Model fit was assessed using the goodness of fit test of four different network statistics (i.e., minimum geodesic distance, indegree, outdegree and edge-wise shared partners statistics) by comparing 100 randomly simulated networks from the ERGM final model to our observed network [25]. Model performance was also assessed by the visual graphical inspection of a random simulated network drawn from the final model. MCMC chain mixing and convergence analysis for each of the parameters were examined by trace plots and marginal density plots. Analyses were carried out in R language [26], using igraph, mice, statnet and coda packages [15, 27–30].

## Results

### Descriptive characteristics of the Georgian pig trade network

A total of 487 questionnaires were conducted between September and November 2012 at four pig rearing regions of the country (Kakheti, n = 120; Shida Kartli, n = 120; Samtskhe-Javakheti, n = 125; Samegrelo Zemo-Svaneti, n = 122). Descriptive results of all sections of the questionnaire are described in Beltran-Alcrudo et al. 2017 (in preparation). Our paper focuses only on the section related to the pig trade.

A total of 599 shipments were reported (578 of which were intraregional and 21 were interregional), involving 163 villages and trading a total of 2,758 pigs (565 replacement sows, 194 boars, 1,888 piglets and 111 ready to slaughter pigs).

Descriptive statistics of pig shipments within regions are depicted in Fig 1A. Regarding both intra and interregional shipments, the median Euclidean distance between shipments was 6.08 km (IQR = 0–13.88 km; max = 328.42 km). The median (95% Confidence Interval) number of total shipments per month was 52 (17.3–86.7). The median (95% CI) number of pigs transported per total shipments per month was 2 (0.6–3.4). The months of March and May registered the highest number of shipments (Fig 1B). These results suggest a local and small pig trade community.

Network visualization (Fig 1C) and network metrics (Table 2) suggest a low density and poorly connected trade community between villages. A close to zero network’s density and global transitivity indicates a poorly connected network. Likewise, the close to zero correlation of the degree values (in- and out- degree assortativity) suggests that degree does not affect the likelihood of trade between villages.

**Table 2:** Network metrics of the pig movement network at the village level in four regions of Georgia.

Network metrics	Value	Meaning
Diameter	9	Greater value of the smallest number of contacts required to connect any two villages of our network [24].
Average path length	3.4	Average distance between all pairs of villages in the network [24].
Density	0.023	Ratio between the number of contacts between villages in the network and the number of all possible contacts, if all villages were to be connected. It measures how intertwined the network is [31].
Indegree assortativity	0.088	Pearson correlation coefficient of the indegree of villages that connect with each other. It measures the tendency of villages to connect with other villages with a similar (or different) indegree [32].
Outdegree assortativity	0.091	Pearson correlation coefficient of the outdegree of villages that connect with each other. It measures the tendency of villages to connect with other villages with a similar (or different) outdegree [32].
Global transitivity	0.088	Proportion of the number of open triads over the number of close and open triads. It measures the tendency of villages to be clustered together, regarding trade connections [31].

### Main drivers associated with pig trade in Georgia

Our final model contained nine statistically significant terms: six village characteristics (edges, region, usage of a middleman to trade live pigs by at least one farmer in the village, usage of a live animal market to trade live pigs by at least one farmer in the village, altitude difference between villages and number of farmers per village) and three structural attributes (mutual, isolates and cyclical ties) (Table 3). All other terms, including the gw*-type terms, were either not significant or lead to a degeneration of the model [21]. Therefore, such terms were dropped and not included into the final model.

**Table 3:** Variables retained and results of the three step ERGM construction process to model the probability of trade among villages in Georgia as a function of village and network characteristics. NA = not applicable; x = node/edge attributes; Xi = vector of node/edge attributes; Si = vector of structural attributes; OR = Odds Ratio; CI = Confidence Interval.

ERGM terms	Null model (m0: edges)	Univariate analysis (m1: edges + x)	Final model (m2: edges + Xi + Si)
	OR (95% CI)	OR (95% CI)	OR (95% CI)
Edges	0.0078	0.00068 (0.0004–0.001)	0.0004 (0.0002–0.0008)
Region	NA	22 (12.7–38.2)	13.6 (7.9–23.50)
Presence Middleman	NA	1.59 (1.29–1.95)	1.5 (1.23–1.82)
Presence LAM	NA	1.82 (1.47–2.27)	1.78 (1.42–2.23)
Altitude difference	NA	0.998 (0.997–0.999)	0.998 (0.9978–0.999)
Farmers per village	NA	1.2 (1.1–1.3)	1.18 (1.09–1.28)
Cyclicalties	NA	NA	1.36 (1.07–1.74)
Isolates	NA	NA	0.15 (0.08–0.31)
Mutual	NA	NA	43 (25–74)
AIC	2402	1838	1661
BIC	2411	1887	1734

Model results confirmed the presence of uniform homophily between regions, meaning that the probability of having a shipment within region is higher than that between regions (Odds ratio (OR) = 13.6; 95% CI = 7.9–23.50). Similarly, other factors were associated with a higher probability of trade, namely, usage of a middleman to trade live pigs by at least one farmer in the village (OR = 1.5; 95% CI = 1.23–1.82), usage of a live animal market to trade live pigs by at least one farmer in the village (OR = 1.78; 95% CI = 1.42–2.23) and having a larger number of farmers in the village (OR = 1.18; 95% CI = 1.09–1.28) (Table 3). These results can only be interpreted considering all other variables in the model remain constant (conditional to the rest of the network).

We observed a slight uniform homophily effect regarding altitude, meaning that villages with similar altitude levels tend to trade more with each other. Reciprocity, measured by the mutual term in the model, is strong and significant (OR = 43; 95% CI = 25–74). This indicates that our network contains more mutual trade ties among villages than expected in a randomly generated network. Similarly, isolated villages with no trade ties tend to happen less often than expected in a randomly generated network (OR = 0.15; 95% CI = 0.08–0.31). The cyclical ties term indicates that, when two villages are connected through the shipment of additional villages (i.e., village A and B are connected through trade happening from A→ Z→ Y →B), there is an increase on the likelihood of direct contact or trade happening between those two villages (i.e., A and B) (OR = 1.36; 95% CI = 1.09–1.28).

**Fig 2:**
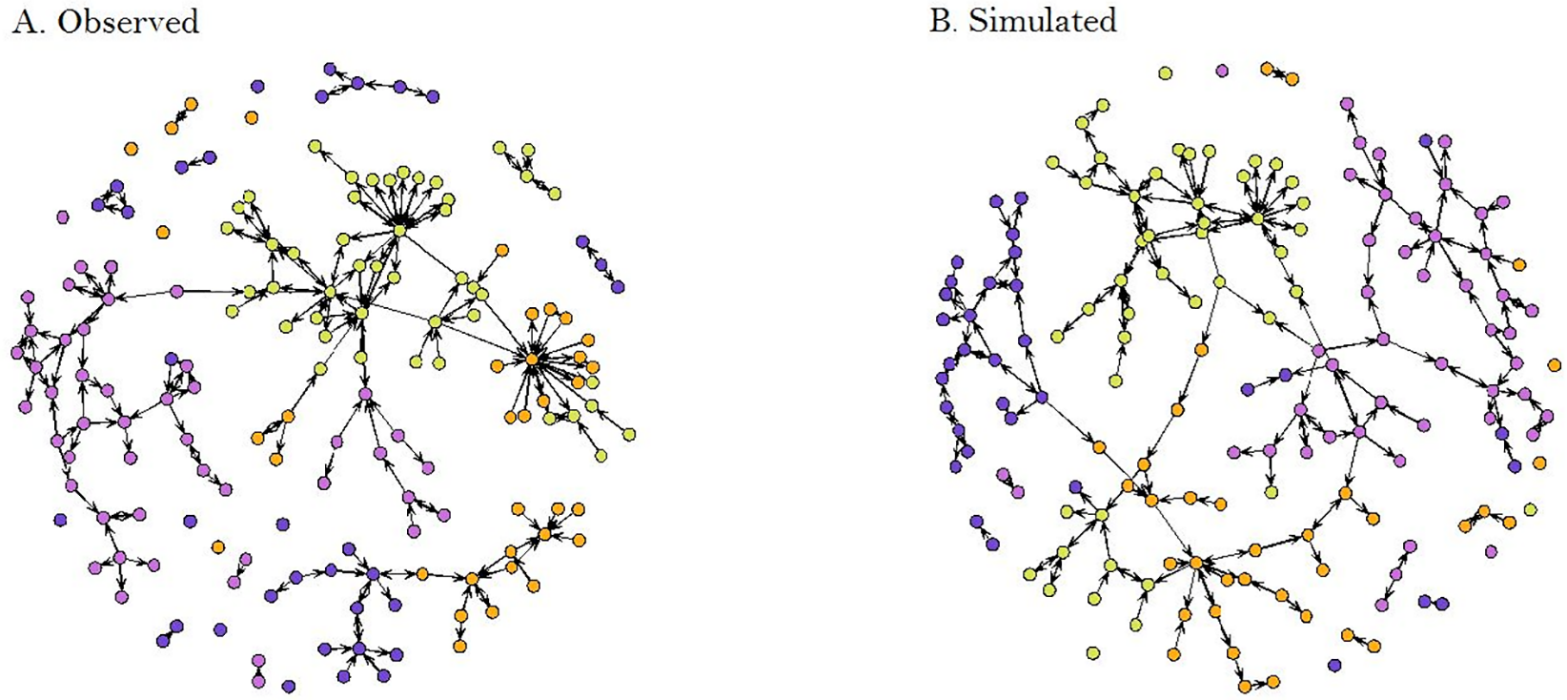
Graphical comparison between the observed (A) and one simulated network (B), obtained through the use of exponential random graph models of the swine trade industry in Georgia, during a twelve-month period. Node coordinates were left fixed for a better visualization of simulated shipments.

**Fig 3:**
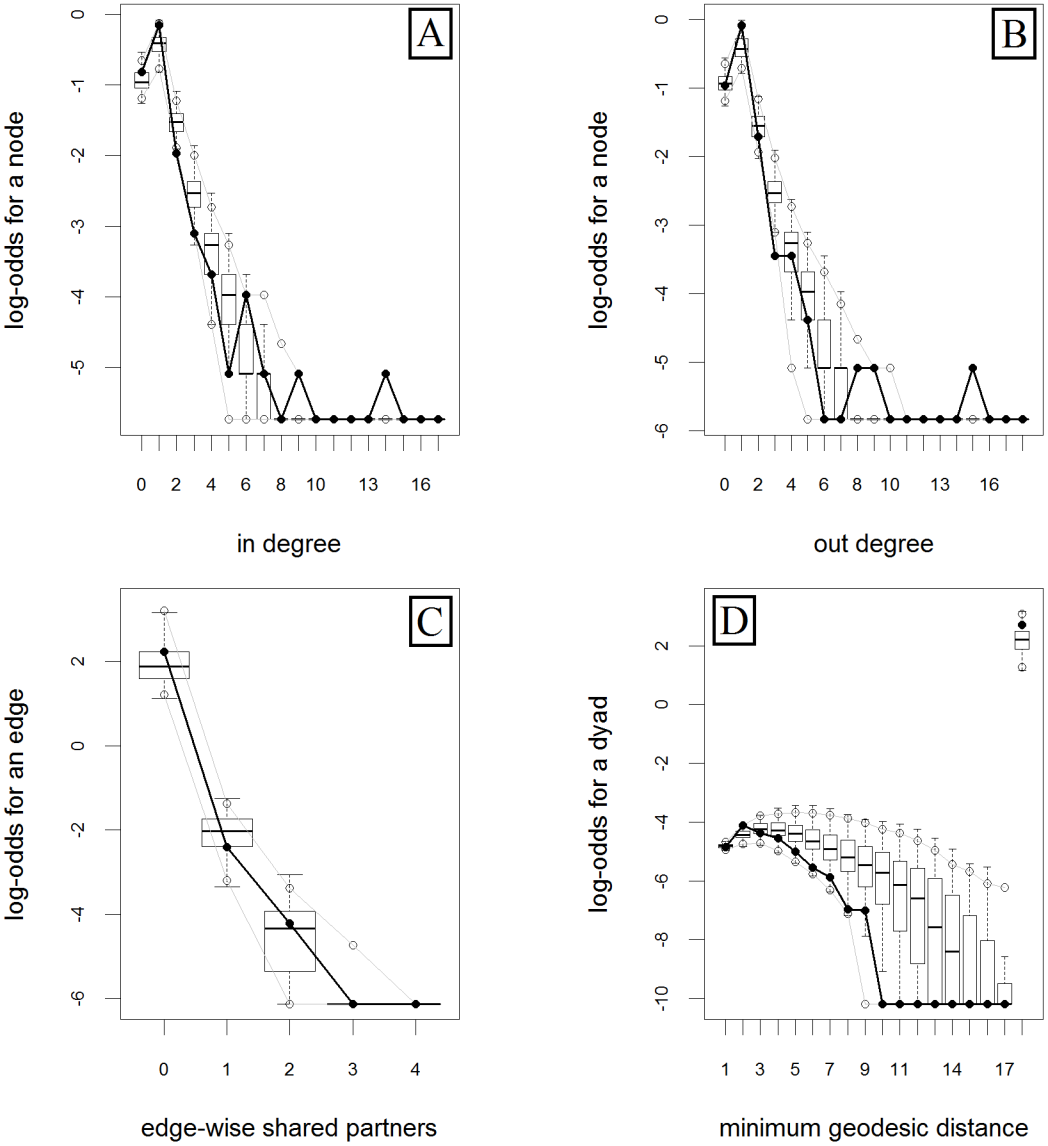
Frequency distribution of the studied goodness of fit diagnostic parameters of the m2 (final) exponential random graph model of the swine trade industry in Georgia, during a twelve-month period. Black lines represent the observed data. Boxplots cover the values of 100 randomly-simulated networks that conform to the model; whiskers represent the 95% CI.

Visual inspection of the graphical output of observed and simulated networks suggest a good fit of the model (Fig 2). This finding is further supported when comparing the indegree, outdegree, edge-wise shared partners and minimum geodesic distance statistics of the observed network with 100 randomly simulated networks that conform to the model (Fig 3). Specifically, the statistics of indegree, outdegree and edge-wise shared partners are generally well captured within the 95% CI of the simulated data (i.e., the observed data is included within the 95% CI distribution of the simulated data) (Fig 3A–3C). However, our model does not perform that well at capturing the minimum geodesic distance, showing a clear overestimation of the minimum geodesic distance values at villages that can be reached by a minimum of 7–12 shipments (i.e., the observed data is out of the 95% CI distribution of the simulated data) (Fig 3D).

**Fig 4:**
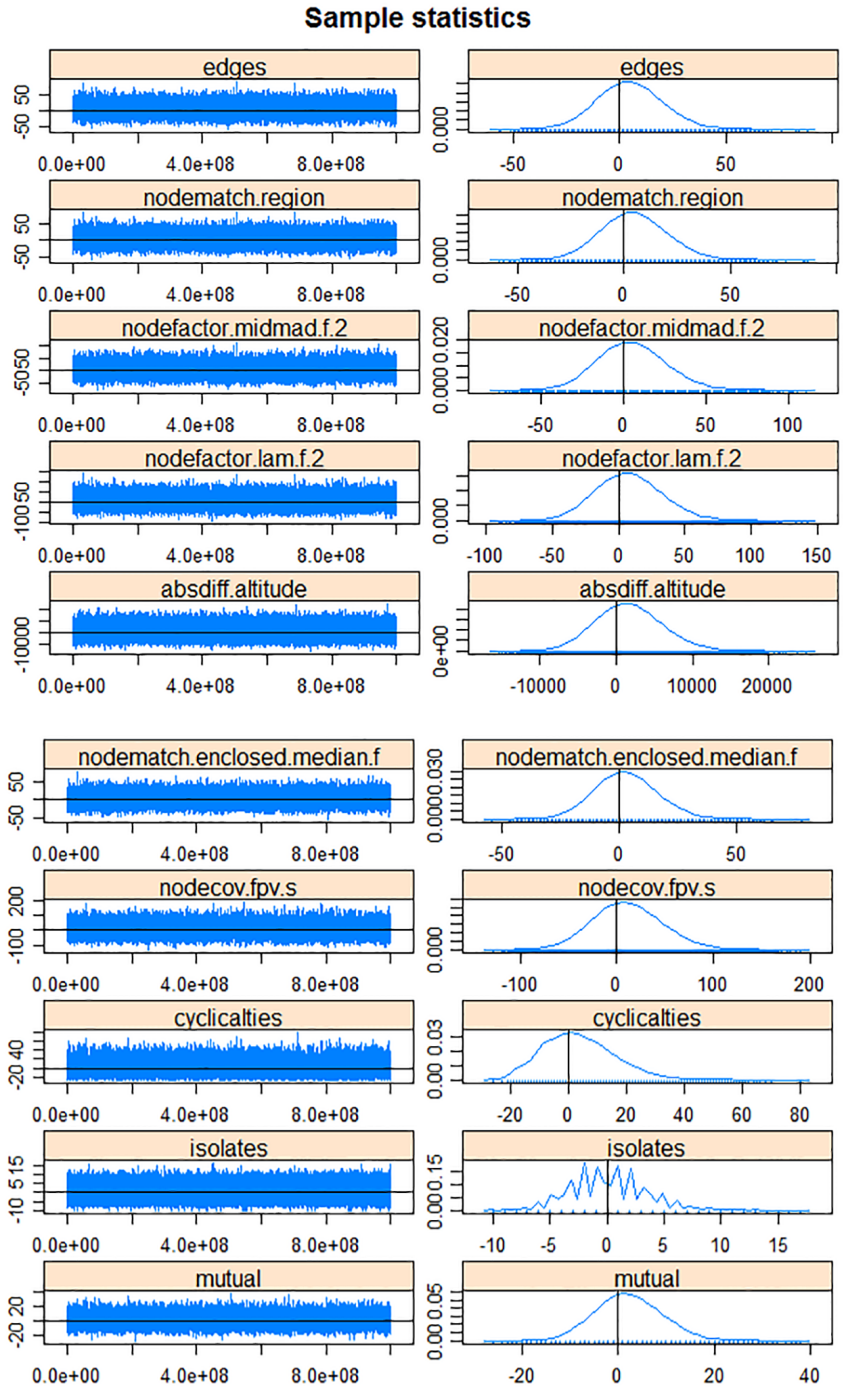
Trace plots (left column) and density plots (right column) of the MCMC diagnostics of the ERGM used to model the probability of trade in the population of villages contained in our four regions of study area in Georgia as a function of both village and network characteristics (m2).

MCMC chain analysis and convergence diagnostics indicates that our model fits our observed network well (Fig 4). For all the MCMC sample statistics, the parameter values are distributed randomly over the observed values (horizontal line set at zero) (trace plot of Fig 4, left column). Moreover, the distribution of the values of the parameters in the MCMC chain (the differences between the observed and simulated values) is approximately normal and centered at zero (marginal density plot of Fig 4, right column). The distribution of “isolates” follows a sawtooth pattern due to its discrete nature.

## Discussion

The present study is the first to describe and characterize the pig trade structure of four pig-rearing regions of Georgia, a country with predominant backyard production systems, for which scarce (if any) data is available on pig trade dynamics and whose pig production was recently devastated as consequence of ASF epidemics. As expected, pig trade was relatively scarce and geographically localized, when compared with other European countries [5, 33] and the United States of America [34]. The main village-related factors contributing to live-pig trade were being from the same region (i.e., local trade), usage of a middleman or a live animal market to trade live pigs by at least one farmer in the village, and having a larger number of farmers in the village. Implementations of strategies for disease prevention and control (i.e., diagnostic testing, clinical inspections, vaccination campaigns) as well as training, outreach and communication activities in villages with those characteristics could be a cost-effective strategy to better prevent and control swine diseases. The near to zero correlation of the degree values (in- and out- degree assortativity) between the connected villages, suggests that degree does not affect the likelihood of trade between villages.

Results from our ERGMs agree with descriptive results in that trade was scarce and local, as expected in most backyard predominant settings [33]. This could explain the limited trade connections between regions (uniform homophily between regions). Nevertheless, our network structurally contains less isolated villages than expected by chance alone, meaning that Georgian villages actively participated in the pig trade even though this trade is sparse and localized. Such localized trade could indicate limited interregional disease transmission through live-pig movements in backyard production systems, as suggested previously [33]. Notwithstanding, when ASF got introduced into the country in 2007, nationwide spread of the disease rapidly occurred, leading to the hypothesis of the important role of indirect transmission pathways (e.g., trade of contaminated pork products that end up being swill fed, the exchange of boar for reproductive purposes, the direct contact of free ranging pigs of different villages, or fomites such as improperly disinfected vehicles or people working in different farms with poor biosecurity measures). Previous studies in backyard pig settings [9, 35] also suggest that, in addition to the normal trade patterns, farmers could attempt to sell their infected pigs to different regions in order to diminish their economic losses. This concept of “emergency sale” was studied by Costard, Zagmutt (9), who concluded that there is a high probability of release of infected pigs into the pig sector through this route, particularly in economically deprived areas, and its consequences should therefore not be overlooked. This would imply that the characteristics of the network discussed in this manuscript are dynamic and thus, subject to change. Costard, Zagmutt (9) also suggested that increasing training and farmers’ awareness is not sufficient to stop emergency sales and associated disease spread; thus, they advocate for financial compensation aimed at culling infected animals and restocking of affected farms (which, unfortunately, is an unfeasible measure in many countries).

The use of a middleman or a live animal market to trade live pigs by at least one farmer in the village significantly increased the probability of trade of that village. Thus, onsite disease control (i.e., diagnostic testing, vaccination campaigns) at live animal markets and increased middlemen disease recognition training and financial compensation could help to control disease spread.

Piglets represent the 68% of all animals moved, with most movements taking place in March and May. This is coherent with the expected production cycle for pigs in the country, whereby most pigs are bought around spring to be slaughtered during the following Christmas period. Results indicate that villages with similar altitude levels tend to trade more with each other than with those located at different altitudes. Contrarily, other studies focused in cattle and small ruminant species [17] suggest higher probabilities of trade between villages located at different altitudes, most probably because of the presence of one central live animal market accessed by most farmers, independently to their distance to it. This difference may also be associated to the different species studied (small ruminant *vs*. domestic pig) and production system used. In our case, as suggested by the small median Euclidean shipment distance, villages trade with nearby neighbors and due to spatial correlation, such villages will encompass similar altitudes. Moreover, this could also be an indicator of lack of social connections with distant areas or lack of infrastructure or measures to transport live pigs to larger distances. A strong and significant reciprocity value supports the idea of a geographically limited trade where social networks amongst neighbors foster a mutual and local relationship for trade. Results of the goodness of fit tests showed that our model globally fit the observed data properly, with the exception of the minimum geodesic distance. During the model building we tried to include structural and edge-level terms in the model to improve the goodness of fit for the minimum geodesic distance statistic; however, these attempts were unsuccessful. This could indicate that our network does not possess a strong structure and thus trade between villages does not follow strict patterns. This could be a result of the above mentioned “emergency sale” concept [9] and seems to be a common trend in backyard production systems, where opportunistic sales leading trade occur, especially when farmers are in need of revenue [36].

However, results of this study must be interpreted with care. Not all regions of the country were included in the questionnaire and the implementation of the questionnaire used a convenience sampling design; thus, the trade network is incomplete and is likely to be over representing the more densely pig populated areas. Additionally, and as a result of the questionnaire structure (i.e., farmers were asked the name of the village they traded with, instead of the specific farmer/entity) data was gathered at the village level, which can lead to some ecological fallacy [37, 38]. Shipments involving villages located in other than our four selected regions (n = 18 shipments) were excluded from the calculation of both network level statistics and centrality measures due to lack of coverage and subsequent data collection at those regions. Missing values regarding the questionnaire answers from villages that were not sampled but that traded with villages that were sampled were imputed, leading to potential bias, smaller standard errors and, potentially, spuriously significant associations. In order to evaluate the potential impact that the imputation of missing values may have had in our results, a second method of imputation (imputation by a random sample from any of the observed values) was used and imputation results of both methods were compared by a Pearson correlation matrix. There was a significant correlation between all tested variables (none of the confidence intervals covering zero, at a CI = 95% level; mean = 0.72, sd = 0.096); therefore, we assumed that the imputation results were robust to be used in the analyses. We also had some limitations associated to the design and digitalization of the survey. For example, a maximum of four entries was established for questions such as the number of buyers/sellers contacted per year. This restriction may lead to truncation of the data, as with no restrictions more buyers/sellers could have been identified. However, the impact of this restriction is likely to be negligible as from the 487 interviewed farmers there were only two farmers reporting four sellers and four farmers reporting four buyers. Even though percentages of response rate per question were initially collected, non-response values were coded as zeros during the data entry procedure, which made impossible to know whether the response was a real “zero” value or a non-response. This could have led to an underestimation of the association between the studied predictive variables and the real probability of trade.

ERGMs are still in the early phases of their development [20] and, although they are increasingly being used in the social scientific literature, they have rarely been used in the veterinary field [17, 33, 39]. We believe ERGMs could be of great value to model complex animal trade networks and to estimate more realistically the probability of disease spread under diverse epidemiological settings. However, due to the above mentioned early stage development of ERGMs, some statistical difficulties still occur in practice, the most important one being model degeneracy [21]. In our specific study, model degeneracy and a poor fit of our model to the observed data regarding the minimum geodesic distance network statistic, prevented us to include interesting structural terms in the model (i.e., gw* terms [20]). Those limitations could partially be solved in the near future as ERGM statistical framework develops and their use expands to other scientific areas. Note that dynamic or unstable networks may be difficult to model, especially if the reasons for their dynamism are illegal practices, for genuine responses regarding those operations may be problematic to obtain.

We hope that the methods and results provides in this study can inform the design of risk-based surveillance and control programs for swine diseases in the country of Georgia. Our data collection and analytical approach could be easily extended and used to other regions where backyard production is abundant and where livestock related information is not frequently collected, allowing for a better understanding of the animal trade dynamics to better prevent and control infectious swine diseases.

## Supporting information

S1 Table Questionnaire. (XLSX)

S2 Table Significant variables in the model. (XLSX)

S3 Table Live pig shipments. (XLSX)
